# Haemosporidian infection risk and community structure determined by duck feeding guild

**DOI:** 10.1017/S0031182025000137

**Published:** 2025-02

**Authors:** Jeffrey A. Bell, Laura E. Bell, Tyler J. Achatz, Kimberly Bates, Riley D. White, Vasyl V. Tkach

**Affiliations:** 1 Department of Biology, University of North Dakota, Grand Forks, ND, USA; 2 Agriculture and Natural Resources Department, University of Minnesota Crookston, Crookston, MN, USA; 3 Department of Natural Sciences, Middle Georgia State University, Macon, GA, USA; 4 Department of Biology, Winona State University, Winona, MN, USA

**Keywords:** Anseriformes, avian malaria, feeding guild, Haemosporida, *Leucocytozoon*, *Parahaemoproteus*, parasite communities, *Plasmodium*, Waterfowl

## Abstract

Birds possess the most diverse assemblage of haemosporidian parasites, although the true diversity is unknown due to high genetic diversity and insufficient sampling across all avian clades. Waterfowl (Order Anseriformes) are an ideal group to discover hidden parasite diversity and examine the role of host ecology in parasite transmission. Waterfowl contain 2 distinct feeding guilds, dabbling and diving, which differ in niche utilization that likely alters vector encounter rates and haemosporidian parasite risk. To determine the role of feeding guild in haemosporidian parasitism we analysed 223 blood samples collected by hunters from the upper Midwest of the United States from 2017 to 2019. Fifty-four individuals were infected by haemosporidian parasites (24·2% prevalence). Infection prevalence differed significantly between dabbling (34·9%, *n* = 109) and diving (14·0%, *n* = 114) ducks. Feeding guild was the only host trait that could predict haemosporidian infection risk, with a significantly higher risk in dabbling ducks. Twenty-four haemosporidian lineages were identified, with 9 identified for the first time. Thirteen lineages were found only in dabbling ducks, 5 only in diving ducks and 6 in both feeding guilds. Community analysis showed that each feeding guild harboured a unique parasite community. There was no phylogenetic signal of feeding guild within a phylogenetic reconstruction of North American waterfowl haemosporidian lineages. Our results demonstrate that waterfowl contain a diverse and distinct community of haemosporidian parasites. The unique composition of each feeding guild determines not only haemosporidian infection risk but also community structure. This is the first report of such an impact for waterfowl feeding guilds.

## Graphical abstract

**Figure:**
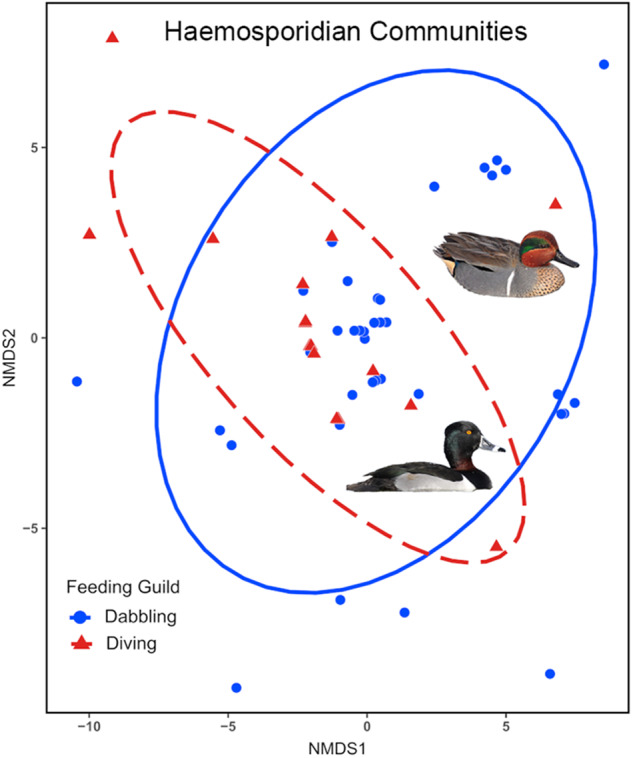

## Introduction

Comprised of the genera *Haemoproteus, Parahaemoproteus, Leucocytozoon* and *Plasmodium*, avian haemosporidians (Apicomplexa, Haemosporida) are a highly diverse and globally distributed group of parasites that infect all avian clades (Valkiūnas, 2005; Clark et al., 2014; Galen et al., 2018a; Fecchio et al., 2020a). However, as sampling has been historically biased towards passerines (Passeriformes), the true diversity of haemosporidians is unknown as recent work on non-passerines has shown they harbour diverse and unique parasites (Bertram et al., 2017; Yabsley et al., 2018; Harl et al., 2022, 2024; Vanstreels et al., 2022). Focused sampling on non-passerine hosts is crucial to not only identify new parasite taxa but also further our understanding of haemosporidian phylogeny (Pacheco and Escalante, 2023).

Avian haemosporidians are protozoan parasites that infect vertebrate blood cells and are transmitted by different haematophagous dipteran vector groups; hippoboscid flies – Hippoboscidae (*Haemoproteus*), blackflies – Simulidae (*Leucocytozoon*), midges – Ceratopogonidae (*Parahaemoproteus*) and mosquitoes – Culicidae (*Plasmodium*) (Valkiūnas, 2005; Santiago-Alarcon et al., 2012; Fecchio et al., 2020a). Variation in avian host traits can alter parasite transmission by impacting vector encounter rates or host immune response to infection (reviewed in Ellis et al., 2020; Fecchio et al., 2020a). Often these effects differ between haemosporidian groups due to differences in environmental factors required for reproduction and development of the different vector groups and the probability of their encounter with vertebrate hosts (Ellis et al., 2020; Fecchio et al., 2020a; de Angeli Dutra et al., 2022). The impact of environmental factors on vector activity may account for the unique haemosporidian parasite distribution patterns observed along altitudinal gradients (Loiseau et al., 2010; van Rooyen et al., 2013; Atkinson et al., 2014; Lotta et al., 2016; Doussang et al., 2019) and the reverse latitudinal diversity gradient found for *Leucocytozoon* (Fecchio et al., 2020b, 2023). Avian groupswith unique traits, such as waterfowl (Anseriformes), can be important systems to understand the effect of host trait variation on haemosporidian infection.

Waterfowl are a diverse and geographically widespread group of birds, yet they are underrepresented in haemosporidian studies (Bensch et al., 2009; Bell et al., 2020; Orlofske et al., 2024). Species of waterfowl harbour 13 named haemosporidian species with only 6 species specific to this host order (Valkiūnas, 2005; Matta et al., 2014). Although there has been a recent spike of interest in the haemosporidian parasites of this group (see Bell et al., 2020; González et al., 2022; Orlofske et al., 2024), Malavi, the largest database of avian haemosporidian genetic data, contains only 216 submissions accounting for 99 different genetic lineages from 43 waterfowl species. This is merely 1·2% of almost 18 000 submissions and 1·9% of more than 5000 genetic lineages within the entire MalAvi database. (http://130.235.244.92/Malavi/index.html, Bensch et al., 2009, accessed in November 2024). The large body size of waterfowl and their reliance on aquatic habitats increases exposure risk to haemosporidian vectors (Meixell et al., 2016; Bell et al., 2020; Orlofske et al., 2024). Additionally, flocking and migratory behaviour both expose birds to greater opportunities for parasite transmission (Matta et al., 2014) and may allow parasite spread across large geographic distances (Levin et al., 2013; Ramey et al., 2015, 2016; Garvon et al., 2016; Meixell et al., 2016).

One of the key ecological traits within waterfowl is feeding guild, which is based on ecological, behavioural and evolutionary differences between dabbling and diving waterfowl (Pöysä and Poysa, 1983; Sun et al., 2017). Dabblers and divers occupy unique ecological niches, differing in foraging strata, diet, nesting location and nest type (Pöysä and Poysa, 1983). Dabblers skim the surface and top water layer for food such as seeds, plants and invertebrates, and can walk easily on land. Even in larger water bodies dabblers spend the majority of their time along the edges at the interface between the aquatic and terrestrial environment. Most dabblers nest on the ground in the surrounding uplands. Divers, in contrast, spend more time away from shore, diving to feed on fish, snails and other invertebrates. Due to diving adaptations, denser bodies and posterior leg position, divers have difficulty walking on land and generally nest near water, often on nesting platforms or in tree cavities. Even in habitats that contain both feeding guilds, their members partition niche space with greater niche overlap within guilds than between them (Pöysä and Poysa, 1983; Pérez-Crespo et al., 2013). Feeding guilds also differ in their sensitivity to changing habitats, as dabblers are more susceptible to changes in water quality and submerged vegetation (Sibilia et al., 2022).

Dabblers are more common than divers in many geographic areas, which could be 1 reason haemosporidian research has focused on dabblers even when divers are included in large survey efforts (Bensch et al., 2009; Meixell et al., 2016; Fleskes et al., 2017). The paucity of focused sampling on divers for haemosporidian identification has limited our understanding of host–parasite dynamics of this group. They likely contain a rich fauna of haemosporidian parasites, like their dabbler counterparts, but are yet unknown. This is illustrated by a recent work focusing on 2 species of diving ducks, greater and lesser scaup (*Aythya marila* and *Aythya affinis*) in Wisconsin (WI), USA (Orlofske et al., 2024). Even with modest sampling they identified 10 lineages infecting diving ducks, including 3 new lineages, and report the first haemosporidian genetic data for three common diving duck species, lesser scaup (*A. affinis*), redhead (*Aythya americana*) and bufflehead (*Bucephala albeola*) (Orlofske et al., 2024). Differences in niche utilization likely alter vector encounter rates and expose each guild to different vector communities, thus altering both risk of haemosporidian transmission and the parasite communities each guild harbours. To date, no study has examined differences in parasite distribution and diversity between waterfowl guilds.

To compare differences in haemosporidian infection risk and community composition, we used molecular methods to screen dabblers and divers collected by American waterfowl hunters from Minnesota (MN), North Dakota (ND) and WI. Birds were collected during the fall hunting season over a 3-year period (2017–2019). We describe the distribution and diversity of haemosporidian parasites within each guild and use statistical and phylogenetic analysis to examine how guild membership may alter infection risk, community composition and phylogenetic relationships of haemosporidian parasites. To our knowledge, our study is the first to examine these differences between feeding guilds in waterfowl.

## Materials and methods

### Sample areas and sample collection

**Figure 1.**
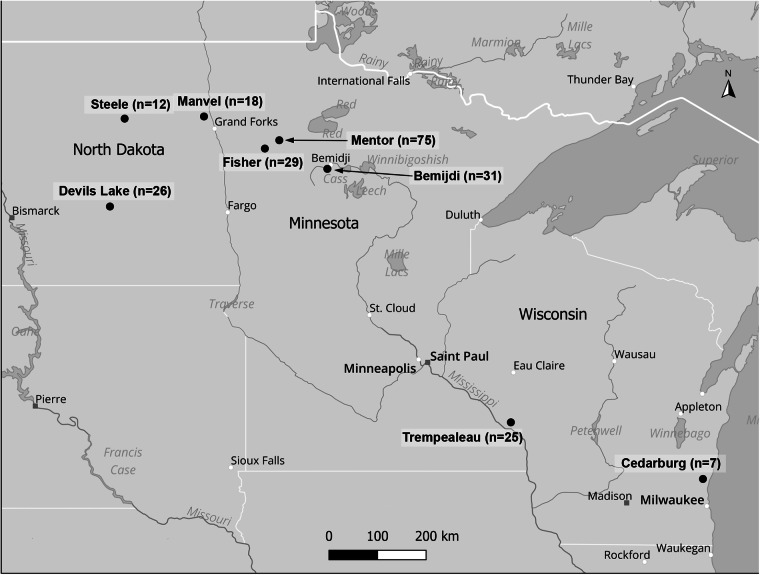
Waterfowl sampling locations in the upper Midwest (QGIS DEVELOPMENT TEAM, 2024).

During the fall waterfowl seasons of 2017–2019, hunters from MN, ND and WI collected blood from the body cavities of 223 harvested waterfowl, representing 8 species of dabblers and 10 species of divers (Table 1). Blood was stored in 95% ethanol and frozen at −20°C for later molecular work. As blood was collected from harvested birds it was not possible to produce blood smears for morphological parasite identification. Hunters provided information on host sampled, sampling location, date of harvest and sex, if possible. Samples were collected from areas associated with the following locations: MN – Bemidji (47° 28′ 25″ N, 94° 52′ 49″ W), Fertile (47° 32′ 04″ N, 96° 16′ 54″ W) and Mentor (47° 41′ 48″ N, 96° 08′ 39″ W); ND – Devils Lake (48° 07′ 47″ N, 98° 52′ 01″ W), Manvel (48° 04′ 22″ N, 97° 10′ 41″ W) and Steele (46° 51′ 24″ N, 99⁰ 55′ 05″ W); WI – Cedarburg (43° 17′ 18″ N, 87° 59′ 15″ W) Trempealeau (44° 0′ 29″ N, 91⁰ 26′ 20″ W). Of the 233 samples collected, 135 were collected in MN, 56 in ND and 32 in WI (Figure 1).

**Table 1.** Distribution of *Leucocytozoon* (Le), *Parahaemoproteus* (Pa), *Plasmodium* (Pl) and total haemosporidian infections in waterfowl collected from the upper Midwest

	No.				Total infected (%)
Host species by feeding guild	Samples	Le (%)	Pa (%)	Pl (%)	
Dabblers					
Northern pintail (*Anas acuta*)	7	2 (28·6)	0 (0·0)	1 (14·3)	3 (42·9)
Northern shoveler (*Anas clypeata*)	6	1 (16·7)	1 (16·7)	0 (0·0)	1 (16·7)^a^
Green–winged Teal (*Anas crecca*)	31	7 (22·6)	8 (25·8)	2 (6·5)	14 (45·2)^b^
Mallard (*Anas platyrhnychos*)	14	5 (35·7)	4 (28·6)	2 (14·3)	8 (57·1)^c^
Wood duck (*Aix sponsa*)	11	1 (9·1)	2 (18·2)	0 (0·0)	3 (27·3)
American wigeon (*Mareca americana*)	1	0 (0·0)	0 (0·0)	1 (100·0)	1 (100·0)
Gadwall (*Mareca strepera*)	15	2 (13·3)	0 (0·0)	0 (0·0)	2 (13·3)
Blue winged teal (*Spatula discors*)	24	3 (12·5)	3 (12·5)	1 (4·2)	6 (25·0)^a^
Total	109	21 (19·3)	18 (16·5)	7 (6·4)	38 (34·9)^d^
Divers					
Lesser scaup (*Aythya affinis*)	12	2 (16·7)	0 (0·0)	2 (16·7)	4 (33·3)
Redhead (*Aythya americana*)	11	1 (9·1)	0 (0·0)	0 (0·0)	1 (9·1)
Ring–necked duck (*Aythya collaris*)	38	4 (10·5)	1 (2·6)	1 (2·6)	5 (13·2)^e^
Greater scaup (*Aythya marila*)	7	3 (42·9)	1 (14·3)	0 (0·0)	4 (57·1)^f^
Canvasback (*Aythya valisineria*)	6	0 (0·0)	0 (0·0)	0 (0·0)	0 (0·0)
Bufflehead (*Bucephala albeola*)	10	0 (0·0)	0 (0·0)	0 (0·0)	0 (0·0)
Goldeneye (*Bucephala clangula*)	9	1 (11·1)	0 (0·0)	0 (0·0)	1 (11·1)
Hooded merganser (*Lophodytes cucullatus*)	11	1 (9·1)	0 (0·0)	0 (0·0)	1 (9·1)
Common merganser (*Mergus merganser*)	8	0 (0·0)	0 (0·0)	0 (0·0)	0 (0·0)
Ruddy duck (*Oxyura jamaicensis*)	2	0 (0·0)	0 (0·0)	0 (0·0)	0 (0·0)
Total	114	12 (10·5)	2 (1·8)	3 (2·6)	16 (14·0)^g^
Grand total	223	33 (14·8)	20 (9·0)	12 (5·4)	54 (24·2)^h^

Coinfections:

a One host with *Leucocytozoon* and *Parahaemoproteus.*

b Two hosts with *Leucocytozoon* and *Plasmodium* and 1 host with *Leucocytozoon* and *Parahaemoproteus*.

c Two hosts with *Leucocytozoon* and *Parahaemoproteus* and 1 host with *Leucocytozoon* and *Plasmodium.*

d Five hosts with *Leucocytozoon* and *Parahaemoproteus* and 3 hosts with *Leucocytozoon* and *Plasmodium.*

e One host with *Leucocytozoon* and *Plasmodium.*

f Two hosts with coinfection of 2 different *Leucocytozoon* lineages.

g One host with *Leucocytozoon* and *Plasmodium* and 2 hosts with dual infections of 2 *Leucocytozoon* lineages.

h Five hosts with *Leucocytozoon* and *Parahaemoproteus*, 4 hosts with *Leucocytozoon and Plasmodium* and 2 hosts with coinfection of 2 different *Leucocytozoon* lineages.

### Molecular identification

DNA was extracted from blood samples using the Qiagen blood and tissue kit (Qiagen, Valencia, California, USA) following the manufacturer’s protocol. DNA extractions were screened by real-time polymerase chain reaction (PCR) to detect haemosporidian DNA. Reactions were carried out using iTaq universal SYBR Green supermix (Bio-Rad, Hercules, California, USA) on a CFX96 real-time thermocycler (Bio-Rad, Hercules, California, USA) using the primers 330 F and 480RL (Bell et al., 2015). We used 15 μL reactions volumes containing 7·5 μL of SYBR Green supermix, 0·6 μL of each primer (10 μM concentration), 3·3 μL of molecular grade water and 3 μL of DNA template (the volume established empirically, approximately 20 ng/μL). The following cycling conditions were used: 95°C for 30 s, followed by 35 cycles of 95°C for 30 s, and 53°C for 35 s (with a plate read) followed by a final melt curve analysis using instrument default settings. All positives determined by real-time analysis were amplified by nested PCR to amplify a 477-base pair (bp) region of the cytochrome b (cyt-b) gene using 2 sets of nested PCR primers. The first set included the initial primers H332F and HaemNR2 with the nested primers H350F and HaemR2 and the second set included the initial primers HaemNFI and HaemNR3 with the nested primers L350F and L890R (Bell et al., 2015). These 2 primer sets together amplify all haemosporidian genera. All nested PCRs were run using OneTaq master mix (New England Biolabs, Ipswich, Massachusetts, USA) in 20 μL reactions on Bio-Rad T100 thermal cyclers (Bio-Rad, Hercules, California, USA). The initial PCR amplifications included 10 μL of OneTaq master mix, 1 μL of each primer (10 μM concentration), 3 μL of molecular grade water and 5 μL of template (the volume established empirically, approximately 20 ng/μL). The nested PCR amplifications differed in using 5 μL of water and 3 μL of PCR product as template. The following protocol was used for all reactions; 95°C for 3 min, then followed by 20 cycles (first amplification)/35 cycles (nested amplification) of 95°C for 30 s, 50°C for 45 s and 68°C for 1 min, followed by a final elongation at 68°C for 5 min. Because of the high sensitivity of nested PCR, negative controls were included in runs to check against possible contamination, although none was found in any PCR runs.

Products from nested PCR amplifications were run on 1·25% agarose gels, stained with ethidium bromide and visualized under ultraviolet light. Positive PCR products were purified using ExoSAP-IT (Affymetrix, Santa Clara, California, USA) and sequenced using BigDye terminator v. 3.1 cycle sequencing kit (Applied Biosystems, Foster City, California, USA) with nested PCR primers (Bell et al., 2015). Forward and reverse sequences were visualized and assembled using Sequencher v. 5.0.1 (Gene Codes Corp., Ann Arbor, Michigan, USA). Chromatograms that showed the presence of multiple infections were scored as coinfections. Coinfections were separated manually following the protocols of Galen et al. (2018b) or by using the program PHASE 2.1.1 (Stephens et al., 2001; Stephens and Donnelly, 2003) following the protocol of Harrigan et al. (2014). Assembled sequences for haemosporidians were aligned using BioEdit v. 7.2.0 (Hall, 1999). A local BLAST (basic local alignment search tool) against the MalAvi database using BioEdit was conducted for all unique haplotypes to identify lineages. As evidence indicates that avian haemosporidian haplotypes differing by 1 cyt-b nucleotide may be reproductively isolated entities (Bensch et al., 2004), we used the conventional practice of referring to each unique cyt-b haplotype as a unique parasite lineage following the standard naming for this group of parasites (Bensch et al., 2009). Sequences were deposited in GenBank (Accession numbers PQ450508 – PQ450553) and the MalAvi database.

### Phylogenetic and statistical analysis

To examine the evolutionary relationships of haemosporidian lineages from North American waterfowl, our newly generated sequences were combined with sequences previously identified from Anseriformes in North America (Orlofske et al., 2024). Only full-length, 477 bp, sequences were used for phylogenetic analysis. *Theileria annulata* (accession number KP731977) served as the outgroup based on its basal position for this group (Galen et al., 2018a).

The GTR + I + G model for base substitution was used for Bayesian inference (BI) reconstruction as determined by jModelTest (Guindon et al., 2003; Darriba et al., 2012). BI analysis was conducted in Mr. Bayes v.3.2.6 (Huelsenbeck and Ronquist, 2001; Ronquist and Huelsenbeck, 2003) and run until the standard deviation of split frequencies stabilized below 0·01. Twenty-five per cent of the resulting trees were discarded as burn-in. Trees were visualized in Figtree (Rambaut, 2009).

All analyses were conducted in program R version 4.4.1 (R core Team, 2024). Chi-square contingency tables were constructed to compare parasite prevalence between feeding guilds implementing Yates continuity correction. Generalized linear models (GLMs) with binomial error distributions to examine the impact of host traits on parasite prevalence were performed using the package glmulti (Clacagno and De Mazancourt, 2010). We modelled the ability of the following traits to predict infection risk; diet (herbivore, insectivore, omnivore, piscivore), feeding guild (dabbling, diving), mass and nest type (upland, cavity, above/near water). Data on diet, nest type and mass were extracted from Elton traits 1.0 (William et al., 2014). We also included collection site as a predictor variable. As host sex was not identified for all samples, we were unable to include this as a predictor variable. Within glmulti, we performed a heuristic search for the best candidate model to explain parasitism based on corrected Akaike information criterion (AICc). The best candidate model, determined by highest AICc model weight (w*_i_*), was used to test the ability of any predictor variable to significantly explain prevalence. Model-average parameter estimates were calculated for each predicator variable, determining their ability to significantly predict risk of infection across all candidate models. We ran 4 separate analyses, 1 for all infections and then 1 for each parasite group independently (*Leucocytozoon, Parahaemoproteus, Plasmodium*).

Differences in haemosporidian communities between feeding guilds were explored using the package vegan (Oksanen et al., 2022). We calculated Jaccard dissimilarity from a matrix of infected hosts, organized by feeding guild, with their corresponding haemosporidian lineage(s). Jaccard dissimilarity measures community dissimilarity ranging from 0 (identical communities) to 1 (dissimilar communities) and is robust against errors due to under sampling (Schroeder and Jenkins, 2018). The resulting Jaccard dissimilarity matrix was used for Permutational Multivariate Analysis of Variance (PERMANOVA) to determine if parasite communities differ significantly between feeding guilds. The analysis was run with 10 000 permutations to determine statistical significance. Prior to the PERMANOVA, we tested for equality in variability (dispersion) within groups using a permutation test with 10 000 permutations. Parasite community structure was further assessed using non-metric multidimensional scaling (NMDS), which uses ordination to visualize community dissimilarity. The analysis uses stress value to gauge the ability of NMDS ordination to accurately represent the dissimilarity between communities. A stress value below 0·05 demonstrates an excellent representation of the data. The R package ggplot2 (Wickman, 2016) was used to construct the NMDS plot.

## Results

Overall, 54 of 223 (24·2%) ducks were infected with haemosporidian parasites with infection prevalence differing significantly between dabbling (34·9% ± 4·6% SE) and diving (14·0% ± 3·3% SE) ducks (*χ*^2^ = 12·06, df = 1, *P* = 0·0005) (Table 1). No infections were found in 4 diving duck species, greater scaup (*A. marila*), bufflehead (*B. albeola*), common merganser (*Mergus merganser*) and ruddy duck (*Oxyura jamaicensis*) (Table 1). *Leucocytozoon* were the most common parasites identified with 33 total infections, followed by *Parahaemoproteus* with 20, and then *Plasmodium* with 12. No *Haemoproteus* parasites were identified in any host (Table 1). Dual infections were found in 11 hosts, 5 hosts with *Leucocytozoon* and *Parahaemoproteus*, 4 hosts with *Leucocytozoon* and *Plasmodium* and 2 hosts with dual infections of 2 different *Leucocytozoon* lineages. Eight of the hosts with dual infections were dabbling ducks (Table 1). Although *Leucocytozoon* and *Plasmodium* prevalence differed between feeding guilds (Table 1), these differences were not significant (*χ*^2^ = 2·72 df = 1, *P* = 0·0992) and (*χ*^2^ = 1·09, df = 1, *P* = 0·2967) respectively. However, *Parahaemoproteus* prevalence was significantly higher in dabbling ducks (*χ*^2^ = 13·11, df = 1, *P* = 0·0003).

In total 24 genetic lineages were identified, 11 *Leucocytozoon*, 4 *Parahaemoproteus* and 9 *Plasmodium* lineages (Table 2). Nine of these lineages were identified for the first time in this study, 4 *Leucocytozoon*, 2 *Parahaemoproteus* and 3 *Plasmodium* lineages (Table 2). Thirteen lineages were restricted to dabbling ducks (13 *Leucocytozoon*, 3 *Parahaemoproteus*, 5 *Plasmodium*), 5 were restricted to diving ducks (3 *Leucocytozoon*, 2 *Plasmodium*) and 6 were found in both feeding guilds (3 *Leucocytozoon*, 1 *Parahaemoproteus*, 2 *Plasmodium*) (Table 2).

**Table 2.** Haemosporidian genetic lineages recovered from hosts examined in this study organized by feeding guild

Feeding guild	Lineage name	Genus	Host(s)	Accession No.
Dabbling				
	AIXSPO02^a^	*Leucocytozoon*	*Anas acuta, Aix sponsa*	PQ450522, PQ450534
	ANAACU01^a^	*Plasmodium*	*Anas acuta*	PQ450521
	ANACRE02	*Leucocytozoon*	*Anas acuta, Anas crecca, Mareca strepera*	PQ450524, PQ450536, PQ450542
	ANACRE05^a^	*Parahaemoproteus*	*Anas crecca*	PQ450533
	ANACYA01	*Plasmodium*	*Anas platyrhnychos*	PQ450537
	ANAPLA01^a^	*Leucocytozoon*	*Anas platyrhnychos*	PQ450508
	ANAPLA02^a^	*Plasmodium*	*Anas platyrhnychos*	PQ450539
	ANAPLA03^a^	*Parahaemoproteus*	*Anas platyrhnychos*	PQ450510
	CYGNUS01	*Parahaemoproteus*	*Anas crecca, Anas clypeata, Anas platyrhnychos, Aix sponsa, Spatula discors*	PQ450514, PQ450526, PQ450532, PQ450538, PQ450544
	DENPET03	*Plasmodium*	*Mareca americana*	PQ450549
	STOCC16	*Leucocytozoon*	*Anas platyrhnychos*	PQ450519
	SW5	*Plasmodium*	*Anas platyrhnychos*	PQ450523
	TUSW05	*Leucocytozoon*	*Anas crecca*	PQ450525, PQ450527
Diving				
	ANSFAB01	*Leucocytozoon*	*Bucephala clangula*	PQ450550
	AYTAFF03^a^	*Leucocytozoon*	*Aythya affinis*	PQ450509
	AYTAFF04^a^	*Plasmodium*	*Aythya affinis*	PQ450516
	AYTCOL01^a^	*Leucocytozoon*	*Aythya collaris*	PQ450541
	MYRMAX01	*Plasmodium*	*Aythya affinis*	PQ450548
Both				
	ANACRE01	*Parahaemoproteus*	*Anas crecca, Aythya marila, Aythya collaris, Spatula discors*	PQ450530, PQ450545, PQ450546, PQ450547
	BT7	*Plasmodium*	*Anas crecca, Aythya collaris*	PQ450529, PQ450540
	BTWE19	*Leucocytozoon*	*Anas clypeata, Aythya affinis, Aythya collaris, Aythya marila, Spatula discors*	PQ450518, PQ450520, PQ450533, PQ450543, PQ450551
	STVAR04	*Plasmodium*	*Anas crecca, Aythya collaris*	PQ450515, PQ450528
	TUSW03	*Leucocytozoon*	*Aythya collaris, Aythya marila, Spatula discors*	PQ450523, PQ450552, PQ450553
	TUSW04	*Leucocytozoon*	*Anas platyrhnychos, Aythya collaris, Aythya marila, Mareca americana*	PQ450512, PQ450513, PQ450517, PQ450525

a Denotes novel lineages identified in this study.

**Figure 2.**
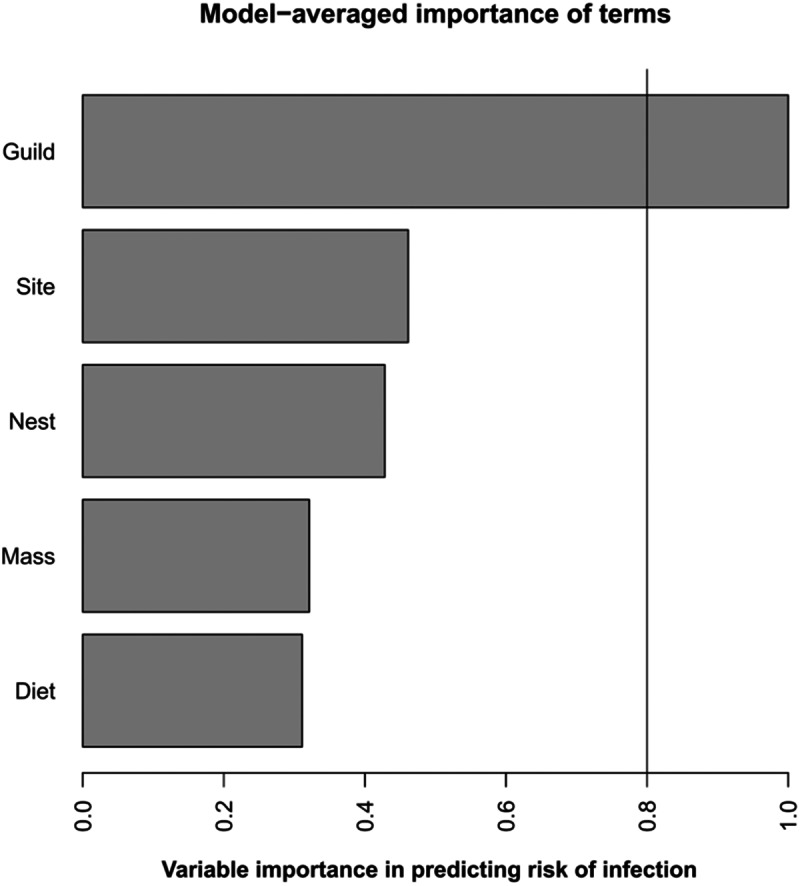
Model average plot of the relative importance of different predictor variables in explaining variation in haemosporidian prevalence. The *x*-axis represents the importance of each variable in predicting haemosporidian infection, variables above the threshold line (0·8) are significant predictors of infection.

Of the candidate models exploring the ability of host traits to predict haemosporidian infection, guild was the only factor included in all models. The best supported model included only guild as an explanatory variable (*w_i_* = 0·1629) and was able to significantly predict haemosporidian infection which was higher for dabbling ducks (*z* = −3·024, *P* = 0·0012). Guild was on the only significant predictor variable across all candidate models (Figure 2). When each parasite group was analysed independently, only for *Parahaemoproteus* were any explanatory variables able to significantly predict infection. The top candidate model contained guild + body mass, + nest type + diet (*w_i_* = 0·2814), with both guild (*z* = −3·030, *P* = 0·0024) and diet (*z* = 2·098, *P* = 0·0359) being significant predictor variables. The probability of *Parahaemoproteus* infection was significantly increased for dabbling ducks and those with a mostly plant-based diet. However, guild was the only significant predictor model across all candidate models.

**Figure 3.**
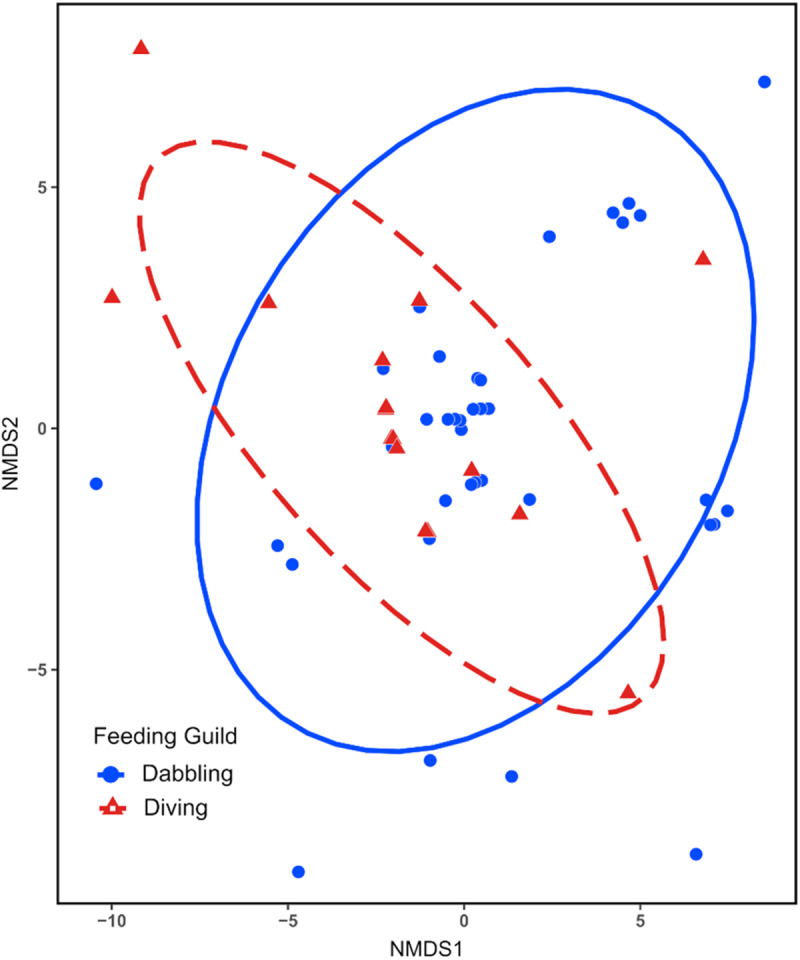
Non-metric multidimensional scaling (NMDS) ordination plot of haemosporidian community dissimilarity between dabbling and diving ducks. Points represent parasite samples within each community surrounded by 95% confidence interval ellipses. The stress value was less than 0·05.

Parasite community composition differed significantly between the feeding guilds (*F*_1,51_ = 2·395, *P* = 0·0032), differences in within community variability (dispersion) do not account for this finding as it was homogenous between the guilds (*F*_1,51_ = 0·117, *P* = 0·7339). NMDS ordination demonstrated parasite community dissimilarity between the feeding guilds (Figure 3). The stress value for NMDS analysis was 0·008, indicating that the ordination well represents the actual dissimilarity between communities.

**Figure 4.**
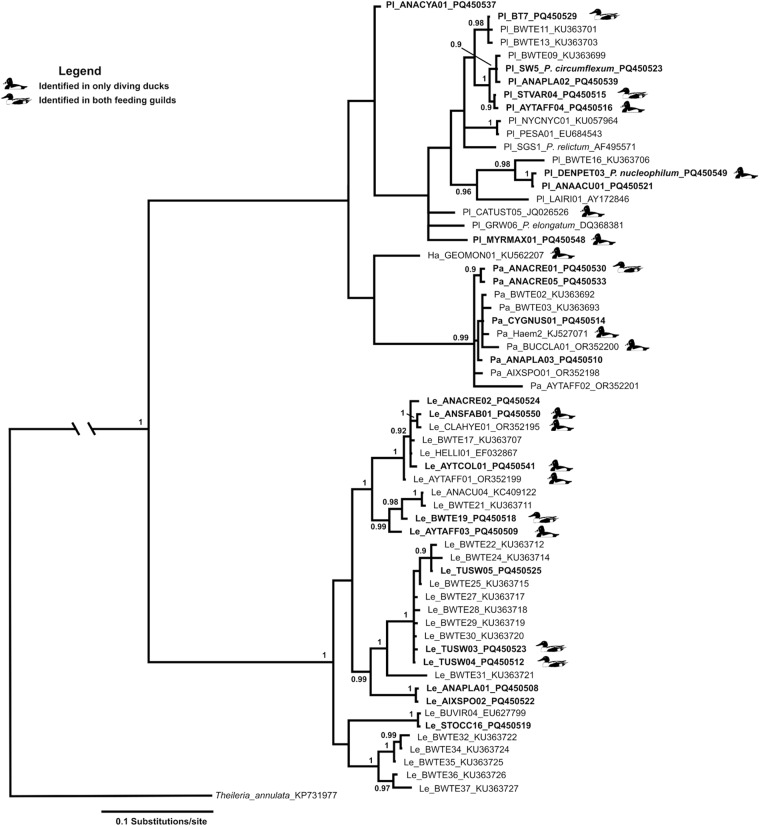
Phylogenetic reconstruction of avian haemosporidians known to infect anseriform hosts from North America. Abbreviations before each lineage identify the taxonomic group (Pl = *Plasmodium*, Ha = *Haemoproteus*, Pa = *Parahaemoproteus*, Le = *Leucocytozoon*) and lineages identified from this study are highlighted. Lineages identified from only diving ducks or found in both feeding guilds are indicated, all unlabelled lineages are identified from only dabbling ducks. Lineages identified in this study are in bold font. Numbers above internodes indicate posterior probability nodal support, with support values lower than 0·9 posterior probability not shown.

Phylogenetic reconstruction demonstrated no pattern of host guild effects on overall tree topology (Figure 4). Lineages both restricted to diving ducks and shared between feeding guilds are dispersed throughout phylogeny, which is dominated by lineages recovered from dabbling ducks (Figure 4).

## Discussion

Differences in ecological niche utilization between diving and dabbling ducks (Pöysä and Poysa, 1983; Pérez-Crespo et al., 2013) expose members of these guilds to differential risks of haemosporidian parasite infection. Of the host traits tested, only guild could explain overall haemosporidian parasitism, with the risk of infection significantly higher in dabbling ducks. The same ecological factors that promote selection of specific sites for breeding and foraging by dabbling ducks also place them at higher risks for parasite transmission by likely promoting increased encounter rates with competent parasite vectors (González et al., 2014; Lutz et al., 2015; Ellis et al., 2020; Fecchio et al., 2020a), thus explaining the higher haemosporidian prevalence and diversity found in dabbling ducks (Table 1). When analysed separately, only *Parahaemoproteus* showed a differential risk between feeding guilds, again higher in dabbling ducks. Although diet was also found to be a significant predictor variable in the best candidate model, this result is due to guild differences as herbivorous ducks had a higher risk of infection and dabblers are mainly herbivorous (Pöysä and Poysa, 1983). As host seeking midges that transmit *Parahaemoproteus* (Valkiūnas, 2005; Santiago-Alarcon et al., 2012; Fecchio et al., 2020a) rely more heavily on visual cues (Bishop, 2002; Bishop et al., 2008), the ecological preferences of dabbling ducks may aid in midges visually locating their hosts for feeding. Dabbling ducks spend most of their time feeding at the water surface along the edges of waterbodies (Pöysä and Poysa, 1983), which increases the amount of time available for vector feeding as compared to diving ducks, which may also explain the higher risk for not only *Parahaemoproteus* but all haemosporidian parasites in dabbling ducks. Additionally, diving may serve as a vector defence mechanism for diving ducks.

Of the 3 genera found, *Leucocytozoon* showed the highest prevalence in both feeding guilds. As *Leucocytozoon* (Galen et al., 2018b; Fecchio et al., 2020b) and their blackfly vectors (McCreadie et al., 2005) are diverse and abundant in the northern latitudes, this provides ample opportunities for parasite transmission to breeding waterfowl. Same environmental factors in northern latitudes that promote *Leucocytozoon* transmission, such as cooler summer temperatures (Fecchio et al., 2020b), may reduce *Parahaemoproteus* and *Plasmodium* transmission for waterfowl, especially for diving ducks. The overall low prevalence of *Plasmodium* is interesting as the water bodies utilized by waterfowl likely harbour high diversity and abundance of mosquitoes that transmit these parasites. The nesting period is key time for haemosporidian transmission (Valkiūnas, 2005) as it provides a sedentary target for blood feeding vectors. For mosquitoes, the high concentration of kairomones, ammonia, carbon dioxide, serve as a strong attractants (Gibson and Torr, 1999; Logan et al., 2010) and promote *Plasmodium* transmission during nesting (González et al., 2014; Lutz et al., 2015; Fecchio et al., 2022), especially in bird species with altricial (i.e. blind, non-feathered) chicks that spend a longer period in the nest prior to fledging and have few defenses against vector feeding. However, the precocial (i.e. alert, feathered) chicks of waterfowl leave the nest once hatched to inhabit wetlands where it is possible that specific microclimatic conditions reduce the concentration of kairomones that mosquitoes rely on for host seeking, thus reducing *Plasmodium* transmission. Additionally, host specificity of mosquitoes may limit transmission (Medeiros et al., 2013) or specific immune responses may exist to reduce *Plasmodium* infections especially as they are the only haemosporidian that reproduce asexually within blood cells giving an additional target for the immune response (Valkiūnas, 2005).

Of the 24 lineages recovered in this study the majority, 18 lineages are restricted to a specific feeding guild, with dabblers showing a high diversity of haemosporidians as compared to divers, 13 versus 5 lineages. The high prevalence of *Leucocytozoon* is matched by a high diversity of *Leucocytozoon* lineages in all groups. Interestingly, however, *Plasmodium* lineages are also rather diverse, with 9 lineages identified despite overall low prevalence. The possibility of mechanisms that promote *Plasmodium* diversity while dampening infection prevalence in waterfowl warrant future studies.

Dabbling and diving ducks support unique haemosporidian communities with limited lineage sharing between communities. The unique species composition, niche utilization and ecological sensitivity of each guild (Pöysä and Poysa, 1983; Pérez-Crespo et al., 2013; Sibilia et al., 2022), determine not only haemosporidian infection risk but also community structure, potentially through different vector exposure. The ability of avian community structure to modulate haemosporidian distribution and diversity has been previously shown at regional (Ellis et al., 2015; Fecchio et al., 2018; de la Torre et al., 2021, 2022), continental (Fecchio et al., 2019) and global scales (Fecchio et al., 2021). Host community composition plays a key role in shaping haemosporidian community dynamics either regardless of or in synergy with climatic and ecological factors affecting vector communities. For example, in eastern North America, Ellis et al. (2015) found that the effects of avian hosts are greater than climatic impacts on haemosporidian communities and de la Torre et al. (2021) found that avian community structure in the Amazonia region of Brazil is a key factor in the community structure of both *Plasmodium* parasites and their mosquito vectors. On the other hand, the synergistic effects of host composition and environmental factors were found to drive haemosporidian community dynamics in South America (Fecchio et al., 2019) and globally (Fecchio et al., 2021). Our results are the first to show that community structure, specifically feeding guilds, impacts haemosporidian distribution and diversity in waterfowl. We demonstrate that avian host community structure likely serves as the main driver of haemosporidian communities in an avian group outside of Passeriformes, the focus of most works on avian haemosporidian research, revealing that the impact of avian composition on haemosporidian assemblages may occur across all avian clades.

Our results do not support the exchange of haemosporidians between dabbling and diving ducks at stopover sites, as most lineages are restricted to each guild and not shared between them with each guild supporting mostly distinct parasite communities. Although annual waterfowl migration has been shown to facilitate parasite transmission across large geographical areas (Levin et al., 2013; Ramey et al., 2015, 2016; Garvon et al., 2016; Meixell et al., 2016), this requires competent vectors existing at stopover sites where multiple species congregate. This is not the case at large stopover areas in the upper Midwest during spring and fall migration as vectors are generally not active due to low temperatures, especially in abundance. Even though species from both guilds eventually reach summering and some wintering areas where vector activity occurs, they again separate to utilize distinct habitats or filter into their respective niches in shared habitats. Thus, differences in utilization of ecological space between feeding guilds are likely the main factor responsible for their distinct haemosporidian communities.

The lack of phylogenetic signal for feeding guild within the overall tree topology of North American Anseriform lineages is likely due to vast under sampling, especially of diving ducks. Our study provides the first genetic data of haemosporidian parasites from 3 common species of diving ducks, canvasback (*Aythya valisineria*), hooded merganser (*Lophodytes cullulatus*) and ring-necked duck (*A. collaris*) and presents only the second reports from five additional common species, lesser scaup (*A. affinis*) redhead (*A. americana*), bufflehead (*B. albeola*), common goldeneye (*Bucephala clangula*) (Orlofske et al., 2024) and ruddy duck (*O. jamaicensis*) (Smith and Ramey, 2015). As dabbling and diving ducks are distinct clades of Anseriformes (Sun et al., 2017), cophylogenetic relationships between feeding guilds and their haemosporidian parasites may exist; however, it can be determined only through additional sampling of diving ducks.

Waterfowl of the upper Midwest support a rich fauna of haemosporidian parasites, which is still not fully described as indicated by the 9 new genetic lineages identified in this study alone. Combined with the 4 new lineages identified recently by Orlofske et al. (2024), this demonstrates that even in a well-studied, economically important group of hosts there is still much to be discovered in terms of their haemosporidian parasites. For example, we report for the first time haemosporidian lineages from northern shoveler (*Anas clypeata*) and American wigeon (*Mareca americana*), 2 common dabbler species, and only the second report from gadwall (*Anas strepera*) (Yang et al., 2021). This highlights the need for focused sampling of historically understudied host groups, specifically avian orders outside of Passeriformes, which hosts most of the genetic lineages of haemosporidians (Bensch et al., 2009). The 39 remaining avian orders (Gill et al., 2024) not only must harbour a significant yet unknown diversity of haemosporidian parasites, but likely also contain distinct clades of parasites as has been demonstrated for Accipitriformes (Yabsley et al., 2018; Harl et al., 2024), Cariamiformes (Vanstreels et al., 2022) and Gruiformes (Bertram et al., 2017). Anseriformes likely contain unique parasite and host relationships (Bell et al., 2020; Orlofske et al., 2024) and potentially unique parasite clades. Additionally, anseriforms are the basal clade of modern birds (Jetz et al., 2012), whose initial radiation began 58–50 million years ago with rapid diversification beginning about 5.3 million years ago (Sun et al., 2017). They could serve as a model to understand parasite and host coevolutionary relationships over different evolutionary scales. They also provide an excellent opportunity to explore the role of cryptic speciation in *Leucocytozoon* parasites, which are abundant and genetically diverse in Anseriformes, yet represented by only a single morphologically identified species, *Leucocytozoon simondi* (Valkiūnas, 2005). To date, no genetic lineage has been linked to *L. simondi* (Bensch et al., 2009) and it likely represents a species complex as has been shown for both *Leucocytozoon toddi* and *Leucocytozoon californicus* in raptors (Harl et al., 2022). The work of Galen et al. (2018b) has shown that *Leucocytozoon* are more speciose than previously thought, due to high levels of cryptic diversity even with low levels of genetic variation in the barcoding region of cyt-b. Future work focused on producing quality blood smears from anseriforms would provide the morphological data necessary to examine the true diversity of *Leucocytozoon* in this group and determine if *L. simondi* does indeed represent a species complex.

Waterfowl can serve as a model system to study haemosporidian parasites in non-passeriform orders as they are widespread, abundant, highly sampled, evolutionarily and ecologically distinct, and support diverse and distinct parasite communities. Sampling haemosporidian parasites of Anseriforms will also aid in elucidating the effect of ecological niche partitioning on haemosporidian parasite communities, as shown herein for diving and dabbling ducks. Future work is warranted, combining morphological and molecular parasite identification with vector sampling to describe haemosporidian parasites and their unique host–vector–parasite relationships within this group. Work on waterfowl can aid in resolving long-standing questions in haemosporidian phylogeny (Galen et al., 2018a) and determine the role of cryptic speciation in their diversification (Galen et al., 2018b; Harl et al., 2022). Unlike most non-passeriform groups where acquiring large sample sizes can be logistically difficult or impossible, waterfowl are captured for banding and harvested by hunters in high numbers every year. Working with local hunters and governmental agencies provides an excellent opportunity to acquire the samples needed to answer these and other questions on the dynamics of haemosporidian parasitism within Anseriformes.

## Data Availability

DNA sequences are deposited in GenBank (PQ450508 – PQ450553). Data on haemosporidian infections have been submitted to the MalAvi database (http://130.235.244.92/Malavi/index.html). Raw data are available by request from the corresponding author.
